# Mobile thrombus of the abdominal aorta: a narrative review

**DOI:** 10.1590/1677-5449.202200282

**Published:** 2022-09-09

**Authors:** Ana Paula Donadello Martins, Leonardo Henrique Bertolucci, Rodrigo Batista Warpechowski, Arthur Angonese, Mariana Saadi de Azevedo, Camilla Rodrigues, Alfredo Augusto Schulte, Silvio Cesar Perini

**Affiliations:** 1 Pontifícia Universidade Católica do Rio Grande do Sul – PUCRS, Porto Alegre, RS, Brasil.; 2 Pontifícia Universidade Católica do Rio Grande do Sul – PUCRS, Hospital São Lucas, Porto Alegre, RS, Brasil.

**Keywords:** abdominal aortic mural thrombus, mural thrombi, aortic mobile thrombus, primary aortic mural thrombus, endovascular therapy, trombo aórtico abdominal, trombo móvel aórtico, trombo mural primário, terapia endovascular, aorta abdominal

## Abstract

A primary aortic mural thrombus (PAMT) is defined as a thrombus attached to the aortic wall in the absence of any atherosclerotic or aneurysmal disease of the aorta or any cardiac source of embolus. It is a rare entity that has high morbidity and mortality. There is no consensus on the ideal treatment of PAMT. The objective of this paper is to review the possibilities for treatment of mobile abdominal aortic mural thrombus. Endovascular therapy and open surgery appear to be the best options for treatment of mobile abdominal aortic mural thrombus. Thus, in patients with favorable anatomy, endovascular therapy is probably the treatment choice, while in those with unfavorable anatomy, open surgery is probably the best option for treatment of a mobile abdominal aortic thrombus. It is important to emphasize that anticoagulation alone can be used as a non-aggressive option and, if this fails, endovascular or surgical methods can then be employed.

## Resumo

O trombo mural aórtico primário é definido como um trombo aderido à parede aórtica na ausência de doença aterosclerótica e/ou aneurismática ou de fonte cardíaca de êmbolo. Trata-se de uma doença rara, porém causadora de alta morbimortalidade, e não há consenso acerca do seu tratamento. Este estudo objetivou revisar as possibilidades na presença de componente móvel. A terapia endovascular e a cirurgia aberta parecem ser as melhores opções, sendo a abordagem endovascular o tratamento de escolha para pacientes com anatomia favorável e a cirurgia aberta o tratamento de escolha para pacientes com anatomia desfavorável. No entanto, a anticoagulação sistêmica apresenta-se como método não invasivo para pacientes com alto risco cirúrgico e como possibilidade terapêutica na falha ou indisponibilidade de abordagem cirúrgica.

## INTRODUCTION

Primary aortic mural thrombus (PAMT) was described for the first time in 1958 by Weissman and Tobin.^1^ It is defined as a thrombus attached to the aortic wall in the absence of any atherosclerotic or aneurysmal disease in the aorta or any cardiac source of embolus. Despite being rare, this type of thrombi has high morbidity and mortality rates, considering that 17% of cases present peripheral embolization and 6% of cases evolve to death.^2^ The first reported case series describing distal embolization caused by aortic thrombus was published in 1981, reporting 20 cases.^3^

The prevalence of PAMT described in the literature is 0.8 – 9.0%.^4^ However, considering that PAMT tends to be asymptomatic until embolization, the true prevalence of the disease is unknown and the increase in reports in recent decades is mainly due to the greater availability of imaging tests.^5^

The pathophysiology behind PAMT in a healthy aorta is unclear. However, in the literature its presence has been associated with prothrombotic disorders like infection,^6^^,^^7^ endovascular manipulation,^7^^,^^8^ closed aortic trauma,^6^^,^^7^^,^^9^^,^^10^ arterial wall tumor,^7^^,^^8^^,^^11^^,^^12^ occult neoplasm,^6^^-^11^,^^13^^-^15 chemotherapy,^7^^,^^16^ hereditary thrombophilias,^3^^,^^6^^,^^7^^,^^9^^,^^17^^,^^18^ essential thrombocytosis,^7^^,^^11^^,^^15^ polycythemia,^6^^,^^11^ antiphospholipid syndrome,^4^^,^^7^^,^^15^ hyperhomocysteinemia,^7^^,^^9^^,^^15^ familial dysfibrinogenemia^7^^,^^15^ and heparin-induced thrombocytopenia.^7^^,^^10^

As previously stated, the clinical presentation of PAMT is usually asymptomatic until embolization occurs.^5^ A review of thoracic PAMT showed that 82% were diagnosed after embolization.^11^ Regarding the prevalence in different genders, the male to female ratio is 1:1^9^ and the average age of diagnosis varies between 40 and 56 years.^10^^,^^14^^,^^15^ The most common embolization sites are the lower limbs, followed by the visceral arteries and the brain.^14^

**Figure 1:**
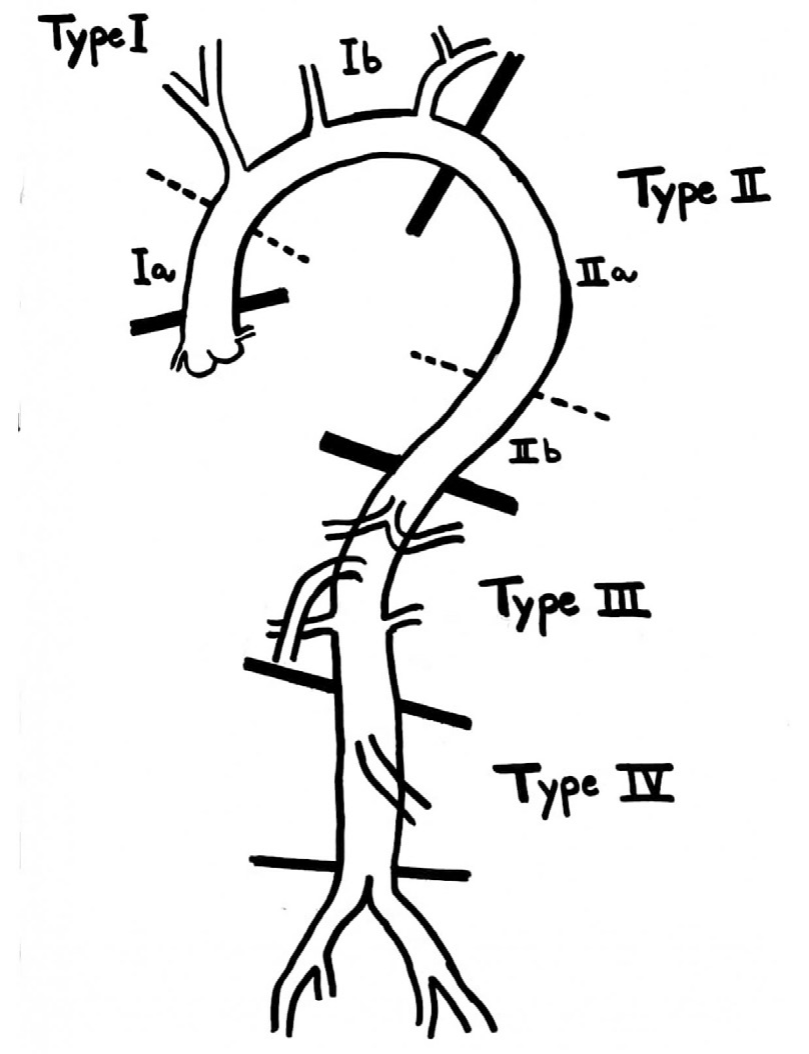
Classification of primary aortic mural thrombus.

Verma’s anatomical classification of PAMT divides it into four types (Figure 1 and Table 1).^14^ In addition to classification by location, these thrombi can also be classified by their morphology. Eccentric or concentric thrombi with no free floating component are classified as Sessile (S). Mural thrombi attached to the aorta proximally with a distal free floating segment of variable length are classified as Pedunculated (P). Complete thrombotic occlusion of the aorta is classified as Occlusion (O).

**Table 1:** Classification of primary aortic mural thrombus.

**PAMT**	**Anatomic location**	**Subclassification**	
**I**	Mural thrombus in ascending and arch of aorta (up to origin of left subclavian artery)	**Ia**	Thrombus limited to ascending aorta
		**Ib**	Ascending aortic thrombus extending into arch or aortic arch thrombus
**II**	Mural thrombus in descending thoracic aorta (distal to left subclavian artery up to coeliac artery)	**IIa**	Descending thoracic aorta thrombus above T8
		**IIb**	Descending thoracic aorta and supraceliac aorta thrombus (T8-L1)
**III**	Mural thrombus in aortic segment between coeliac artery to lowest renal artery		
**IV**	Thrombus between lowest renal artery to aortic bifurcation		

There is no consensus on the ideal treatment for PAMT. The objective of this narrative review is to evaluate the best treatment for mobile abdominal aortic thrombi classified as types III or IV.

## METHODS

### Inclusion criteria

All articles that enrolled subjects with mobile thrombus of the abdominal aorta and described their management were included.

### Exclusion criteria

Studies involving just the thoracic aorta were excluded.

### Literature search

Independent electronic searches were performed by two reviewers (AM and LB) on the following databases: MEDLINE (via PubMed), EMBASE, Cochrane CENTRAL, ERIC, and SciELO.

The search strategy used for Medline (PubMed) was as follows: mobile aortic thromb* AND (treatment OR management) and primary aortic mural thromb* AND (treatment OR management). These terms were adapted for use on other databases in accordance with their specific features or requirements.

No restrictions on language or date of publications were applied for any of the databases. The reference lists of all studies included were hand searched to identify potential data sources.

### Selection of studies

Eligibility of studies was assessed by two authors (AM and LB). Titles and abstracts were screened for inclusion. Following that, studies that seemed fit for inclusion and studies that could not be categorized due to lack of information in the abstract underwent full text screening by the same authors.

The few disagreements that occurred during the study selection process were mainly resolved by consensus.

## RESULTS

Treatment options for aortic thrombi described in the literature include primary disease anticoagulation, thrombolysis, thromboaspiration, surgical thrombectomy, and endovascular or open surgery to exclude the thrombus from the aorta (Tables 2 and 3). However, these reports are limited and the best treatment for each case and for prevention of peripheral embolization is not well established.

**Table 2:** Articles evaluating treatment of abdominal aortic mural thrombus with anticoagulation.

**Author**	**Year**	**Type of publication and level of evidence (Oxford Centre)**	**Location of thrombus**		**Treatment**	**Follow-up**	**Outcomes**
Hahn et al.^19^	1999	Case reports (6 patients) - Level 4	Not reported		Anticoagulation	0-12 months	1- Lost to follow-up. 1-Noncompliant: multiple episodes of thrombosis/ embolus. 4- Uneventful; no recurrence.
Bowdish et al.^20^	2002	Retrospective review (5 patients - 4 abdominal aorta) - Level 4	3 suprarenal and 1 infrarenal		Anticoagulation	29+/-11 months (median, 16 months)	Uneventful; no recurrence.
Poirée et al.^21^	2004	Case reports (2 patients) - Level 4	Not reported	Anticoagulation		2 weeks - 3 months	Uneventful; no recurrence.
Fayad et al.^9^	2013	Meta-analysis (200 patients - 28 abdominal aorta) - Level 3a	Not reported	112 patients: anticoagulation.		Not reported	Not reported
Caron and Anand^22^	2017	Case report - Level 4	Supra and infrarenal aorta	Anticoagulation		12 months	Uneventful; no recurrence.
Reyes Valdivia et al.^23^	2017	Retrospective study (8 patients - 4 abdominal aorta) - Level 4	3 infrarenal. 1 visceral aorta	Anticoagulation		Median: 23 months	Uneventful; no recurrence.
Patrício et al.^24^	2018	Case report - Level 4	Visceral aorta	Anticoagulation		6 months	Uneventful; no recurrence.
DeKornfeld et al.^25^	2018	Retrospective study (6 patients - 1 abdominal aorta) - Level 4	Infrarenal	Anticoagulation		Not reported	Not reported.

**Table 3:** Articles evaluating the surgical and endovascular treatment of abdominal aortic mural thrombus.

**Author**	**Year**	**Type of publication**	**Location of thrombus**	**Treatment**	**Follow-up**	**Outcomes**
Reber et al.^26^	1999	Prospective study (8 patients - 4 abdominal aortic thrombus) - Level 4	Not reported	Transabdominal endarterectomy	4-24 months (median: 13 months)	Uneventful; no recurrence
Dougherty et al.^27^	2000	Case reports (2 patients) - Level 4	Infrarenal	Catheter-directed thrombolysis (urokynase therapy). 1 patient: further anticoagulation.	54 and 36 months.	Uneventful; no recurrence.
Bosma et al.^28^	2007	Case report - Level 4	Infrarenal aorta	Aortotomy and selective thromboembolectomy of all crural vessels.	Not reported	Not reported
Zhang et al.^29^	2008	Case report - Level 4	Infrarenal aorta	Endovascular stent grafts.	9 months	Uneventful; no recurrence
Luckeroth et al.^30^	2009	Case reports (2 patients) - Level 4	Infrarenal aorta	Endovascular placement of covered stents.	36 months	Uneventful; no recurrence
Kim et al.^31^	2011	Case report - Level 4	Pararenal aorta	Hybrid surgery using wire-directed balloon catheter thrombectomy.	5 months	Uneventful; no recurrence
Fayad et al.^9^	2013	Meta-analysis (200 patients - 28 abdominal aorta) - Level 3a	Not reported	88 patients: surgical treatment (endovascular treatment excluded)	Not reported	Not reported
Verma et al.^14^	2014	Retrospective study (19 patients - 9 abdominal aorta) - Level 4	1 visceral aorta, 2 infrarenal aorta	Visceral aorta: trapdoor aortic thrombectomy. Infrarenal aorta: 1 aortobiiliac embolectomy and 1 aortobiiliac embolectomy and subsequent endovascular stenting.	> 6 months	Trapdoor thrombectomy: 1- Minimal residual sessile thrombus on CT. No recurrence Complete recovery from paraplegia and renal failure. Infrarenal aorta: Uneventful; no recurrence.
Kadoya et al.^32^	2018	Case report - Level 4	Infrarenal aorta	Endovascular stent grafts.	12 months	Uneventful; no recurrence.
Murter et al.^33^	2019	Case reports (3 cases) - Level 4	1- Visceral aorta. 2- Infrarenal aorta. 3- Visceral and infrarenal aorta.	Percutaneous thrombectomy.	1 month	Uneventful; no recurrence .
Borghese et al.^34^	2020	Retrospective study (9 patients - 5 abdominal aorta - 3 pedunculated) - Level 4	2 visceral aorta. 1 infrarenal aorta.	1: open balloon thrombectomy followed by surgical aortic bypass. 2: aortic bypass.	22 months	No deaths.

Systemic anticoagulation is a widespread treatment for aortic thrombus in general. Tunick and Kronzon describe an important decrease in mortality in patients with aortic thrombi with a mobile component, without evidence of a reduction in embolic events. However, the need for more robust studies is emphasized.^35^ Saric and Kronzon^36^ highlight the paucity of data regarding all therapies and the benefits or not of anticoagulation.

Caron and Anand^22^ reported a case treated successfully with warfarin anticoagulation. This choice was made because of impaired renal function, which is a relative contraindication for endovascular therapy. Use of systemic anticoagulation is also strongly supported by Hahn et al, Bowdish et al., Patrício et al., Valdivia et al. and Poirée et al., whose patients didn’t present thrombus recurrence or re-embolization. The surgical approach was only indicated for patients who do not respond to conservative treatment.^19^^-^21^,^^23^^,^^24^

Regarding the choice of anticoagulation drug, most studies used unfractionated heparin (UFH) for a short period of time and then switched to warfarin,^20^^,^^22^^,^^24^^,^^25^ targeting an international normalized ratio (INR) between 2 and 3^20^^,^^25^ or 2.5 and 3.5.^24^ The duration of treatment remains uncertain and should be individualized, ranging from 4 months to lifelong for patients with a history of hypercoagulable state.^19^^,^^20^ One patient was maintained on 100 mg aspirin alone after a warfarin regimen.^25^

One patient received UFH for 24-48 hours, followed by low molecular weight heparin (LMWH) for 1 month, then acenoucumarol to maintain INR in the range of 2-3.^23^ One patient used enoxaparin and the target dose used was based on antifactor Xa level, 0.3 to 0.8 units.^20^

However, a high rate of thrombus recurrence and maintenance by conservative therapy was observed in a review study published in 2019, which thus recommended endovascular surgery for type IV thrombi and exploratory laparotomy for type III.^5^

A systematic review with meta-analysis about thrombi in general – including thrombi of the thoracic and the abdominal aorta – reported 28 of 200 patients with abdominal aortic thrombus and 176 with a mobile component. In that study, anticoagulation was used for 112 patients and this subset presented more thrombus recurrence, more thrombus maintenance, and more major limb amputations than the surgical group, with 25% requiring surgery later. Treatment, complications, and recurrence were not reported by location type of the thrombus and the study did not include endovascular or thrombolytic therapies.^9^

Two cases of type IV mobile aortic thrombus treated with thrombolytic therapy were described in 2000. One patient had the catheter positioned in a popliteal thrombus, but the aortic thrombus remained unchanged after 12 hours of continuous infusion. She was then prescribed anticoagulation because she refused a surgical procedure. Warfarin was discontinued after 6 months and 54 months later the patient remained asymptomatic and without thrombi recurrence. The second patient was successfully treated with thrombolytic therapy, using a catheter directed to the aortic thrombus, but developed massive colonic bleeding. No additional anticoagulation or surgery was needed at 36 months of follow-up. Both patients were treated with urikynase.^27^

There is no evidence for use of antiplatelet agents and statins for prevention of embolism.^35^

Some cases with different surgical approaches are mentioned in the literature. Of these, as early as 1999, Reber et al. reported four cases effectively treated with transabdominal endarterectomy, with no evidence of new embolic events or recurrence of thrombus over 4-24 months of follow-up. The authors suggested operative approaches are the best option if the patient presents with low surgical risk.^26^ A similar result was observed by Bosma et al. in a case of infrarenal thrombus with embolization to the right kidney, left deep femoral artery, and lateral branches of the inferior mesenteric artery in a patient with elevated factor VIII. In this case, major amputation, hemicolectomy, and crural vessel thrombolysis were associated with transabdominal thrombectomy and life-long use of warfarin.^28^

In a retrospective study of patients with a history of distal embolization, three cases of primary abdominal aortic thrombus with a mobile component were found, 1 type III and 2 type IV. For the patient with PAMT type III, trapdoor aortic thrombectomy was used to treat visceral aorta involvement in the emergency setting with full recovery and no recurrence. To treat the type IV thrombi, in one patient aortoiliac embolectomy was performed and in the other exclusion by stent graft was achieved using a 16x12x70 mm Excluder limb (W. L. Gore, Flagstaff, Ariz). They had 6 month-follow-up without complications.^14^

Although recent, endovascular therapy is promising and has low rates of recurrence and re-embolization. The first case reported in the literature was performed in 2008 in a patient with a descending thoracic aortic thrombus and infrarenal aortic thrombus. A staged procedure was planned to reduce the risk of complications. The thoracic thrombus was treated first, because of the higher risk of embolization. Then, one week later, the infrarenal thrombus was excluded using a bifurcated Gore Excluder abdominal aortic stent graft (Gore-Tex, W. L. Gore & Assoc., Flagstaff, Ariz.). After nine months of follow up, the patient remained free from complications.^29^

In the following year, 2 cases were reported by Luckeroth et al., treated using a 20 mm x 3.75 cm AneuRx aortic cuff (Medtronic, Minneapolis, MN) and post dilation with a 27 mm XXL balloon (Boston Scientific, Natick, MA) for one case and a 16 mm x 14 mm x 7 cm Gore Excluder contralateral leg endoprosthesis (WL Gore, Flagstaff, AZ) and post dilation with a 14 x 40 mm percutaneous transluminal angioplasty (PTA) balloon for the other case, both with distal embolectomy. In these cases, balloon exclusion of the contralateral iliac artery was performed during the procedure to reduce the risk of embolization. Follow-up was for 36 and 6 months, respectively, and both patients remained asymptomatic.^30^

Endovascular surgery seems to be an option when conservative treatment isn’t successful, as observed in a patient with antiphospholipid syndrome treated with 100 mg of aspirin plus 75 mg of clopidogrel and heparin. Kadoya et al. describe use of three 40-mm-long stents (Palmaz XL stent; Cordis, Milpitas, California) with good results after 12 months of follow-up.^32^

Furthermore, Borghese et al. recently reported three cases of mobile aortic thrombus. Treatment of an infrarenal thrombus was attempted with anticoagulation plus antiplatelet agent, but subsequent surgical placement of a 16x40 mm stent and 8 x 37 mm biiliac bare metal stent express (Boston Scientific, Natick, Mass) was needed because of thrombus maintenance. For treatment of type III thrombi, one patient underwent aortic bypass and the other received a prosthetic aortic and renal by-pass after unsuccessful open balloon thrombectomy. Both were maintained on antiplatelet therapy.^34^

In addition, hybrid surgical treatment using wire-directed balloon catheter thrombectomy was reported in a patient who had undergone a complex surgical procedure 17 days earlier and received an initial UFH infusion. In this case, it was necessary to conduct mechanical thrombectomy using a Trerotola device (Arrow International Inc.) with a 5F rotating nitinol basket fragmentation cage, because of the resistance of the thrombus to the balloon.^31^

Regarding percutaneous thrombectomy, a series of 3 cases was described in 2019. One patient had a visceral mobile aortic thrombus, 1 had infrarenal aorta involvement, and the third had involvement of both segments, with 2 mobile thrombi. A continuous aspiration system was used (Indigo mechanical thrombectomy system; Penumbra, Alameda, Calif) to perform the thrombectomy, in combination with real-time intravascular ultrasound (IVUS) guidance. Patients were followed for 1 month and had no residual thrombi, recurrence, or new thrombotic events.^33^

## DISCUSSION

The most frequently reported type of PAMT was type II (38%). The literature on PAMT type III and IV is scarce. The narrative, systematic, and meta-analysis articles published cover treatment of PAMT as a single entity and do not segment it by affected area. This review was conducted with the aim of elucidating the treatment options for mobile abdominal aortic thrombus.

Anticoagulation is considered the therapy of choice by many authors and vascular surgeons. The anticoagulants of choice were low-molecular-weight heparin or unfractionated heparin, later switching to warfarin at hospital discharge. There is one case of thoracic mobile thrombus treated with direct oral anticoagulants with success^33^ and one case of a sessile thrombus of aorta that was treated with embolectomy. At hospital discharge, direct oral anticoagulant was prescribed with complete resolution after 2 years.^37^

The meta-analysis published by Fayad et al.^9^ does not differentiate between treatment for thrombi of the thoracic or abdominal aorta and excludes endovascular treatment. In the comparison of anticoagulation versus surgical treatment, surgical treatment demonstrated benefits in the outcomes thrombus persistence and recurrence, distal embolization, complications, and limb loss. Thus, surgical treatment seems to be a superior option to use of anticoagulants for treatment of PAMT.

Regarding endovascular treatment in patients with abdominal aortic thrombus, there are no studies comparing endovascular approaches with open surgical treatment. The evidence that currently exists for endovascular treatment derives from case reports and retrospective studies. The endovascular approach seems to be the best option in cases of infrarenal and pararenal abdominal aortic thrombus. All cases treated with endovascular therapy had favorable outcomes.^14^^,^^29^^-^32 It is important to emphasize that endovascular treatment has been increasingly used for thoracic aortic thrombus and appears to be an effective and safe option.^29^^,^^38^^-^43 Studies carried out to date do not report differences between use of stent grafts and uncoated stents.^11^^,^^14^

A meta-analysis of treatment strategies for patients with descending thoracic aortic mural thrombus evaluated 74 patients, 24 of whom were treated with anticoagulation, 19 with open surgery, and 29 with endovascular therapy. Of these, 6 patients who underwent open surgery and 9 patients who were managed with anticoagulation presented with recurrence. Eight of the recurrence patients were treated with endovascular approaches (4 from the open surgical group and 4 from the anticoagulation group). There were 4 deaths in the anticoagulation group and 2 deaths in the endovascular group. There were no recurrences in the endovascular group.^11^ There are no meta-analyses comparing anticoagulation, open surgery, and endovascular approaches in the abdominal aorta.

There are a few reports of thoracic aortic thromboaspiration^44^^-^46 and abdominal aortic thromboaspiration^33^ with favorable outcomes. As studies advance, in the future this may become an option for treatment of abdominal aortic thrombus and may even be considered an option for treatment of mural aortic thrombus.

Endovascular therapy and open surgery seem to be the best options for treatment of abdominal aortic thrombus. Studies evaluating the thoracic aorta indicate that endovascular therapy is now the first line treatment. Despite this, endovascular therapy relies on adequate anatomy without prohibitive clot loading. Thus, in patients with favorable anatomy, endovascular therapy is probably the therapy of choice, while in those with unfavorable anatomy, open surgery is probably the best option. It is important to emphasize that anticoagulation alone can be attempted and, if unsuccessful, an endovascular or surgical approach can then be employed.
